# Expert consensus on digital restoration of complete dentures

**DOI:** 10.1038/s41368-025-00388-2

**Published:** 2025-07-30

**Authors:** Yue Feng, Zhihong Feng, Jing Li, Jihua Chen, Haiyang Yu, Xinquan Jiang, Yongsheng Zhou, Yumei Zhang, Cui Huang, Baiping Fu, Yan Wang, Hui Cheng, Jianfeng Ma, Qingsong Jiang, Hongbing Liao, Chufan Ma, Weicai Liu, Guofeng Wu, Sheng Yang, Zhe Wu, Shizhu Bai, Ming Fang, Yan Dong, Jiang Wu, Lin Niu, Ling Zhang, Fu Wang, Lina Niu

**Affiliations:** 1 State Key Laboratory of Oral & Maxillofacial Reconstruction and Regeneration, National Clinical Research Center for Oral Diseases, Shaanxi Key Laboratory of Stomatology, Department of Prosthodontics, School of Stomatology, The Fourth Military Medical University, Xi’an, China; 2 State Key Laboratory of Oral Diseases, National Clinical Research Center for Oral Diseases, West China Hospital of Stomatology, Sichuan University, Chengdu, China; 3 Shanghai Stomatological Hospital & School of Stomatology, Fudan University, Shanghai, China; 4 National Center for Stomatology & National Clinical Research Center for Oral Diseases & National Engineering Research Center of Oral Biomaterials and Digital Medical Devices, Department of Prosthodontics, Peking University School and Hospital of Stomatology, Beijing, China; 5 State Key Laboratory of Oral & Maxillofacial Reconstruction and Regeneration, Key Laboratory of Oral Biomedicine Ministry of Education, Hubei Key Laboratory of Stomatology, School & Hospital of Stomatology, Wuhan University, Wuhan, China; 6 Zhejiang Provincial Clinical Research Center for Oral Diseases, Key Laboratory of Oral Biomedical Research of Zhejiang Province, Engineering Research Center of Oral Biomaterials and Devices of Zhejiang Province, Department of Prosthodontics, Stomatology Hospital, School of Stomatology, Zhejiang University School of Medicine, Hangzhou, China; 7 Hospital of Stomatology & Guanghua School of Stomatology & Guangdong Provincial Key Laboratory of Stomatology, Sun Yat-sen University, Guangzhou, China; 8 School and Hospital of Stomatology, Fujian Medical University, Fuzhou, China; 9 Institute of Stomatology, School and Hospital of Stomatology, Wenzhou Medical University, Wenzhou, China; 10 Department of Prosthodontics, Beijing Stomatological Hospital, Capital Medical University, Beijing, China; 11 Guangxi Key Laboratory of Oral and Maxillofacial Rehabilitation and Reconstruction, College of Stomatology, Guangxi Medical University, Nanning, China; 12 Department of Stomatology, Air Force Medical Center, Beijing, China; 13 Shanghai Engineering Research Center of Tooth Restoration and Regeneration & Tongji Research Institute of Stomatology & Department of Prosthodontics, Stomatological Hospital and Dental School, Tongji University, Shanghai, China; 14 Nanjing Stomatological Hospital, Affiliated Hospital of Medical School, Institute of Stomatology, Nanjing University, Nanjing, China; 15 Chongqing Key Laboratory of Oral Diseases, Chongqing Municipal Key Laboratory of Oral Biomedical Engineering of Higher Education, College of Stomatology, Chongqing Medical University, Chongqing, China; 16 Department of Prosthodontics, School and Hospital of Stomatology, Guangdong Engineering Research Center of Oral Restoration and Reconstruction & Guangzhou Key Laboratory of Basic and Applied Research of Oral Regenerative Medicine, Guangzhou Medical University, Guangzhou, China; 17 Key Laboratory of Shaanxi Province for Craniofacial Precision Medicine Research, Clinical Research Center of Shaanxi Province for Dental and Maxillofacial Diseases; Department of Prosthodontics, College of Stomatology, Xi’an Jiaotong University, Xi’an, China

**Keywords:** Removable prosthodontics, Oral manifestations

## Abstract

Digital technologies have become an integral part of complete denture restoration. With advancement in computer-aided design and computer-aided manufacturing (CAD/CAM), tools such as intraoral scanning, facial scanning, 3D printing, and numerical control machining are reshaping the workflow of complete denture restoration. Unlike conventional methods that rely heavily on clinical experience and manual techniques, digital technologies offer greater precision, predictability, and efficacy. They also streamline the process by reducing the number of patient visits and improving overall comfort. Despite these improvements, the clinical application of digital complete denture restoration still faces challenges that require further standardization. The major issues include appropriate case selection, establishing consistent digital workflows, and evaluating long-term outcomes. To address these challenges and provide clinical guidance for practitioners, this expert consensus outlines the principles, advantages, and limitations of digital complete denture technology. The aim of this review was to offer practical recommendations on indications, clinical procedures and precautions, evaluation metrics, and outcome assessment to support digital restoration of complete denture in clinical practice.

## Introduction

Edentulism is a growing concern in prosthodontics.^1^ The number of edentulous patients worldwide had risen substantially over the last three decades. This rapid surge in edentulism has been attributed to a rise in aging population. The trend is expected to continue over the next 20 years.^2^ Apart from the loss of teeth, edentulism has a profound impact on daily life, affecting chewing, swallowing, and speech. It also contributes to alveolar bone resorption and temporomandibular joint disorders. Collapse of the lips due to missing teeth also creates a sunken facial appearance that adversely affects social interactions and psychological well-being.^2–7^ Edentulism has become a major public health challenge.

Complete dentures are commonly used for restoring oral function and appearance in edentulous patients. These removable, mucosa-supported prostheses replace missing maxillary and/or mandibular teeth and associated structures. They directly impact the quality of life of patients as well as their physical and mental well-being.^8^ However, conventional denture fabrication is a complex and technique-sensitive process. Patients often require multiple visits from the initial consultation to the final fitting,^9^ which can be especially challenging for elderly individuals.^10^ The process also comes with technical hurdles such as distortion of impression materials,^11^ inaccuracy in occlusal records,^12^ and potential human errors during fabrication.^13–15^ These factors can compromise the fit, function, and esthetics of the dentures, making it difficult to achieve optimal results. Managing more complex cases with conventional methods is even more challenging that requires repeated adjustments and prolonged treatment time.

New opportunities for complete denture construction have emerged with the rapid advancement of digital dentistry.^9,10,16,17^ Technologies such as intraoral scanning, facial scanning, and computer-aided design and computer-aided manufacturing (CAD/CAM) have brought denture fabrication into the digital age. Unlike traditional methods, digital workflow offers advantages from acquiring preliminary impressions and designing custom trays to fabricating personalized dentures. These include better clinical efficiency, improved denture accuracy, reduced human error, a shorter production timeline, and a more comfortable patient experience.^9,18–20^

Despite these benefits, there is still no unified standard for applying digital technologies in complete denture fabrication. Clinicians often face inconsistencies in case selection, workflow implementation, and treatment outcome evaluation. Because of the growing adoption and significant potential of digital denture technology, there is an urgent need to establish standardized protocols, enhance clinical effectiveness, and further develop this field. This expert consensus on digital complete denture restoration is the result of extensive research and discussion. This expert consensus is a collaborative effort by specialists from prosthodontics and clinical experts across the nation based on the current clinical evidence and the collective wisdom of leading authorities in digital complete denture rehabilitation. The consensus seeks to provide clinicians with clear guidelines on standardized procedures, case indications and contraindications, operational protocols, and quality control measures. By refining these aspects, the goal is to improve restoration quality, minimize complications, and ultimately enhance treatment outcomes and the overall quality of life for edentulous patients. In particular, this review aims to offer practical recommendations on case indications, clinical procedures and precautions, evaluation metrics, and outcome assessment, thereby supporting the effective implementation of digital complete denture fabrication in clinical practice.

## Indications and case selection

This consensus applies to edentulous patients undergoing digital complete denture restoration. Apart from streamlining the fabrication process, digital technologies can expand the indications for complete dentures to include:

1. *Patients with difficulty tolerating conventional impressions*. Individuals with a strong gag reflex, anxiety, or fear of conventional impression techniques may benefit from intraoral scanning, which offers a non-invasive alternative that reduces psychological stress and improves treatment comfort.^21,22^
2. *Patients with high esthetic expectations*. Digital tools enable precise control over tooth arrangement and morphology through CAD software. This technology provides a customized design guided by three-dimensional facial esthetics to better meet the esthetic goals of the patients.^23–25^
3. *Patients who need a faster treatment process*. Those requiring complete dentures within a short timeframe due to work or social commitments can benefit from digital workflows, which can reduce the traditional five-appointment process to just two or three visits.^9^
4. *Patients with complex intraoral conditions requiring diagnostic or therapeutic dentures*. Digital technologies enable the rapid fabrication of dentures for assessment and adjustment. Stored digital data also allows easy replication of existing dentures to helping to shorten or even eliminate the edentulous period.^26,27^
5. *Patients facing geographical or logistical challenges*. The ability to transmit and store digital data remotely implies that dentures may be designed and fabricated in different locations, reducing the need for frequent clinic visits. This is particularly beneficial for patients in remote areas, those with transportation difficulties, or those with limited time.^9^
6. *Patients at high risk of denture loss or damage*. With digital archiving, lost or damaged dentures can be easily and accurately reproduced. This helps minimize inconvenience and the need for extensive refitting.^28,29^

## Digital clinical workflows

### Acquisition of digital information

#### Digital edentulous models

These models may be obtained through intraoral or extraoral scanning. Intraoral scanning is a practical option for most patients, especially for those with a strong gag reflex or difficulty tolerating conventional impression techniques.^30^ It is also beneficial for patients with limited mouth opening that hinders the insertion of stock trays.^31^ However, extraoral scanning may be preferable in cases where patients have excessive mobility of oral soft tissues, significant undercuts, or uncontrollable saliva secretion.^32–34^ Clinicians should carefully evaluate anatomical factors (such as alveolar ridge shape and soft tissue mobility), patient-related factors (including mouth opening and gag reflex), and the accuracy and reliability of the scanning technology when determining the most appropriate scanning method.

1. Intraoral scanning (direct scanning)
  Intraoral scanning captures three-dimensional data of the edentulous alveolar ridge and surrounding soft tissues using an intraoral scanner. However, achieving high-quality images can be challenging due to the smooth, featureless nature of the edentulous mucosa, which lacks stable anatomical landmarks. Other difficulties include saliva production, tongue movement, and the continuous positional changes of the mucosal tissue. Current research^11,35–38^ suggests that intraoral scanning is more accurate in the maxilla than in the mandible. For maxillary edentulous patients with sufficient alveolar ridge height and adequate attached mucosa, definitive models may be obtained through intraoral scanning. For patients with compromised maxillary or mandibular edentulous ridges, particularly those with extensive mobile mucosa or significant interference from tongue movement, intraoral scanning alone may not be sufficient for denture fabrication. These patients often require a secondary impression.^39–45^
2. Extraoral scanning (indirect scanning)
  Extraoral scanning begins with the process of obtaining edentulous impressions using conventional techniques. These physical impressions or their resulting plaster models are then scanned to create digital models. The procedure follows standard intraoral protocols for conventional edentulous impressions. The protocols include selecting an appropriate stock tray and impression material to ensure proper border extension, and capturing all essential anatomical landmarks for an accurate and complete impression. Once the physical impression is obtained, it undergoes extraoral scanning to generate a precise digital model that preserves the details necessary for denture fabrication.

#### Facial scanning

Facial scanning uses non-contact scanning technology to capture detailed facial features and create three-dimensional images. These images serve as a valuable reference for the esthetic design of complete dentures. This technique is particularly beneficial for edentulous patients with noticeable dentofacial deformities or those with high esthetic expectations, as it enables precise recording of facial features for a more personalized denture design.^46^

Facial scanning is usually performed in two positions: full-smiling and postural.^47,48^ Since edentulous patients lack anterior teeth for registration, scanning is also conducted while the patient wears a registration device. A facebow fork is commonly employed for this purpose, which provides a stable extraoral registration area, often enhanced with geometric markers, along with an intraoral tray that attaches securely to the maxilla using impression material.^48–50^ Once scanning is complete, the three-dimensional files should be exported in formats compatible with digital design software. Common formats include Stereolithography Language/Standard Triangle Language/Standard Tessellation Language (STL), Object File Format (OBJ), and Polygon File Format (PLY). Among these, OBJ and PLY formats are particularly useful for esthetic design because of their ability to incorporate color texture information. This feature renders these formats more popular in clinical applications.

### Digital design and manufacture of custom tray complexes

A custom tray complex is a personalized combination of a custom tray and auxiliary structures. It is designed to accommodate the unique anatomical characteristics of a patient’s edentulous ridge. The primary function of a custom tray complex is to enable precise capture of definitive impressions and accurate recording of jaw relations. The core component is the tray body, which is supported by auxiliary structures such as tissue stops, occlusion rims, and gothic arch tracers. All of these components contribute to stability, proper alignment, and functional accuracy during the denture fabrication.

#### Custom trays

Because of the limitations in intraoral scanning accuracy for edentulous arches, the two-stage impression technique remains the gold standard for edentulous impressions.^51^ A custom tray must be designed and fabricated prior to obtaining the definitive impression. In CAD software, the process begins with importing a digital edentulous model, followed by marking anatomical landmarks as guided by the software. The model is then virtually analyzed, undercuts are filled, and tray borderlines are defined to generate the tray body. Next, tray handles are designed and integrated with the tray body, completing the digital design of the custom tray. The final design is then saved in STL format for further processing and fabrication.^52,53^

#### Tissue stops

Tissue stops are strategically placed protrusions on the intaglio surface of a custom tray. These stops are designed to ensure direct contact with the underlying tissues when the tray is seated. Direct contact enhances stability, improves accuracy during impression taking, and helps maintain a uniform thickness of the impression material.^54–56^ Properly designed tissue stops should support precise tray positioning without obstructing the flow of impression material or compromising impression accuracy. The custom tray design is usually imported into reverse engineering software, where tissue stops of the desired shape are integrated into the corresponding areas of the intaglio surface. For added flexibility, detachable tissue stops can be incorporated using a “mortise and tenon” structure that permits easy adjustment as needed.

#### Occlusion rims

Integration of digital technology enables the simultaneous design of occlusal rims and custom trays to create a monolithic occlusion rim-custom tray complex. When digital edentulous models, preliminary jaw relation records, and facial scan data are obtained during the initial visit, these datasets can be aligned using anatomical landmarks during custom tray design. This patient-specific information helps guide the concurrent design of the occlusion rim to ensure a more precise and efficient workflow.

In cases where facial scanning is not performed and only preliminary jaw relation records are available, clinicians can still establish reference points such as the midline, commissural line, and occlusal plane. These markers can then be digitized using an intraoral scanner. The captured digital information can serve as a reference for designing the occlusion rim with greater accuracy.

#### Personalized gothic arch tracers

Gothic arch tracing is a well-established method for recording centric relation. Digital technology has introduced new ways to enhance its design, fabrication, and clinical application. A gothic arch tracer usually consists of three components: a registration pin, two registration plates, and a fixing plate. The registration plates are attached to the maxillary or mandibular occlusion rim. In many cases, gothic arch tracers are integrated with a custom tray. This integration enables definitive impression taking and centric relation recording to be completed in a single step.^57,58^

One of the registration plates is designed with a smooth surface to facilitate the movement of the registration pin, while the other registration plate and the fixing plate help secure the pin in place. The pin is positioned to align with the registration plate of the opposing jaw to ensure precise tracing. The design should also incorporate space for vertical adjustment of the pin to accommodate clinical modifications.

#### Digital manufacture of custom tray complexes

After completing the digital design of the custom tray body and its associated structures (e.g., tissue stops, occlusion rims, gothic arch tracers), all data should be exported in STL formats. 3D printing technology is generally recommended for fabricating custom tray complexes.^52–54,57–60^ Commonly used printing materials include light-cured resins and polylactic acid.^61–63^

### Definitive impressions and recording of jaw relations

#### Fabrication of definitive impressions using custom trays with tissue stops

The custom tray should first be tried-in intraorally. If a detachable tissue stop is included in the design, it should be assembled before proceeding. Next, the tray margin should be checked intraorally, and any overextended edges carefully adjusted. For trays with detachable tissue stops, border molding is performed by applying silicone to the custom tray margin and intaglio surface, excluding the tissue stop areas. The tray is then accurately re-seated intraorally to complete border molding. The thickness of the tissue stops ensures that the intaglio surface of the tray maintains a uniform space for the impression material.

Once the silicone has polymerized, the tray is removed, the tissue stops are also removed extraorally, and light-body silicone is loaded into the tray. The tray is then reinserted, ensuring a precise fit guided by the polymerized putty silicone. The patient is instructed to perform border molding movement again to capture the definitive impression.

For trays with non-detachable tissue stops, the exposed areas are examined extraorally after completing border molding under their guidance. A slow-speed handpiece (5000–20,000 rpm) is used to trim any necessary regions. Finally, light-body silicone is applied to fabricate the definitive impression. Any excess impression material overflowing onto the outer surface of the tray is carefully trimmed to avoid interference with vertical dimension assessment and occlusal stability.

#### Recording of jaw relations

1. General recording methods
  After the impression-making process is completed, the maxillary and mandibular custom trays are inserted into the patient’s mouth. The occlusion rims are evaluated, and necessary adjustments are made to establish the ideal occlusal plane and lip support.^12^ The patient is then instructed to retract the mandible and occlude naturally so that centric relation may be recorded. To prevent mandibular protrusion, a spherical bulge is incorporated into the post dam area of the maxillary custom tray. The patient is guided to slowly close the jaw while gently licking the spherical bulge, facilitating mandibular retrusion. Alternatively, swallowing is encouraged, or gentle pressure is applied to the patient’s chin to assist in proper mandibular positioning.^64^
  Once the ideal vertical dimension and a stable, repeatable horizontal relationship are confirmed, occlusal record material is injected between the maxillary and mandibular occlusion rims to capture the occlusal relationship. After the bite registration material has polymerized, the midline and commissure line are marked intraorally. This step can be replaced by acquiring facial scanning data of the patient wearing the custom tray complexes.
2. Determining jaw relation with gothic arch tracer
  If obtaining centric relation using the conventional method is challenging, gothic arch tracing with a 3D-printed device may be used to assist in determining jaw relation after the ideal vertical dimension has been established.^57,58^
  To perform this procedure, the 3D-printed registration plate and registration pin are first assembled with the custom tray extraorally. While maintaining the established vertical dimension, the registration pin is installed and adjusted so that it lightly contacts the maxillary registration plate. The patient is then instructed to repeatedly perform protrusive and lateral movements. During these movements, the mandibular trajectory is traced by the registration pin onto the registration plate, forming a characteristic “gothic arch” shape. The apex of the gothic arch is identified as the patient’s centric relation position. Once the apex is determined, the fixing plate with a fixation screw is attached over the center of the pinpoint tracing. Finally, bite registration material is injected between the maxillary and mandibular occlusal rims to record the occlusal relationship.
3. Determining jaw relation with jaw motion tracking system
  The jaw motion tracking system is a device that employs ultrasonic, optical, or other technologies to record mandibular movement trajectories in three-dimensional space. By capturing the patient’s functional mandibular movement patterns, it provides essential data for diagnosis, treatment planning, and the design and fabrication of prostheses.^64–67^
  The components of a jaw motion tracking system (i.e., signal receiver, maxillary registration plate, mandibular bite fork, and signal transmitter) must first be assembled prior to use. After ensuring proper connections, jaw registration begins by using the maxillary registration plate to establish the relationship between the maxillary occlusal plane and the head. The mandibular bite fork is then attached to the labial side of the anterior region of the mandibular occlusion rim to capture mandibular movements. Once installation is complete, the signal transmitter is connected to the mandibular bite fork. Following the software prompts, the patient is guided through a series of protrusive and lateral movements. The recorded mandibular movement parameters can then be exported for virtual articulator transfer.
  The jaw motion tracking system can also be integrated with muscle function analysis to sequentially record the patient’s habitual position, retracted position, closed position during small-range opening and closing movements, and gothic arch apex position. This data allows for the calculation and prediction of most repeatable mandibular position of the patient for accurate determination of centric relation.^67^

### Digital conversion and transfer of jaw relations

#### Acquisition of digital definitive models and other multimodal data

The definitive impression, adjusted occlusion rims, and interocclusal records are scanned to convert them into digital information, which is then exported in STL format. If facial scanning was performed during the definitive impression stage, the data should be exported in PLY or OBJ format. If mandibular movement was recorded, the maxillary registration fork with the bite record should also be scanned and exported in STL format. In addition, digital facebow parameters and mandibular movement trajectory data for virtual articulator transfer are exported from the jaw motion tracking system.

#### Transfer of jaw relations

Transferring jaw relations is a crucial step in complete denture restoration to ensure accurate recording and transfer of the spatial relationship of the patient’s jaws to the articulator. This step provides a reliable foundation for tooth arrangement and occlusal adjustment. The integration of digital technology has streamlined what was traditionally a complex process. This consensus highlights three digital methods for transferring jaw relations of edentulous casts: (1) digital transfer to an average-value articulator, (2) transfer to a personalized virtual articulator based on facial scanning, and (3) transfer to a personalized virtual articulator based on digital facebow data.^67^

1. Transfer to average-value virtual articulator
  An average-value articulator is a mechanical device pre-configured with standard anatomical parameters. If only the interocclusal relationship of the edentulous jaws is recorded during jaw relation registration, the digital models of the edentulous arches along with the custom trays and occlusion rims can be imported into dental CAD software. The virtual average-value articulator is activated once the occlusal registration of the digital models is complete. The mandibular occlusal rim is then adjusted to align with the average occlusal plane position of the articulator, and the models are manually “mounted” onto the virtual articulator. Using preset average parameters of the software, mandibular movements of the patient can be simulated in the virtual environment.^68^
2. Transfer to personalized virtual articulator based on facial scanning
  This method utilizes facial scan data to position edentulous models in the articulator by aligning stable anatomical landmarks such as the infraorbital point and the upper margin of the external auditory canal with articulator structures. This enables a more individualized simulation of the maxillary position relative to the head.^19,68^
  Compared to average-value articulators, this approach provides better accuracy in occlusal plane angulation and incorporates patient-specific facial esthetics. However, it does not directly transfer complex mandibular movement data such as condylar trajectories. Instead, it enhances virtual tooth arrangement and occlusal design.
  Before scanning, anatomical landmarks should be marked on the patient’s face. The digital edentulous models are then registered and aligned with the facial scan of the patient wearing the custom tray in CAD software. The reference plane of the articulator such as the Frankfort horizontal plane is identified and aligned with the maxillary frame. By positioning the hinge axis points with the condylar balls of the articulator, a simple personalized virtual articulator is created.^67^ Once mounted, the registered edentulous models can simulate mandibular movements in the virtual space.
3. Transfer to personalized virtual articulator based on digital facebow data
  This method utilizes a jaw motion tracking system to accurately record the relationship between the patient’s maxilla and head while also capturing mandibular movement trajectories. The collected data is then imported into CAD software for the construction of a highly-customized virtual articulator.^69,70^
  To implement this approach, the jaw motion tracking system records the anatomical position of the maxilla relative to the head and detailed mandibular movements. The edentulous models, digital facebow data, and mandibular movement data are then imported into CAD software. Transfer to the virtual articulator is accomplished using feature point matching for precise simulation of mandibular function.

### Digital design of complete dentures

#### Denture bases

In dental CAD software, the design process usually begins with identification of anatomical landmarks and defining borderlines. The software automatically generates preliminary borderlines based on these markers, which clinicians can adjust as needed. The denture base should extend as much as possible to improve stability while ensuring it does not interfere with physiological movement. Its thickness must strike a balance between strength and patient comfort. Once the initial denture base is generated, labial and buccal support can be modified based on the patient’s facial profile, age, and gender to restore natural contours and esthetics.

#### Arrangement of artificial teeth

1. Esthetic design
  The arrangement of maxillary anterior teeth is central to the esthetic design of complete dentures. Using intraoral and facial scanning data, a 3D virtual patient may be constructed to provide a precise foundation for digital tooth arrangement and optimizing esthetic outcomes.^19,25,68^
  In dental CAD software, teeth are matched to the arch form, and the position of the maxillary anterior teeth is adjusted relative to the incisive papilla and canines to simulate age-related characteristics. Facial scan data captures upper lip morphology, including lip lines in postural and smiling positions, for assessment of incisal edge exposure. For patients with severe maxillary resorption and facial collapse, denture base thickness can be modified to restore labial support.
  Digital tooth arrangement can reference tooth databases to customize a patient’s facial profile, age, skin tone, and arch size. Pre-extraction photographs or data from well-fitting existing dentures can also be incorporated. The three-dimensional virtual patient further enhances doctor-patient communication, allowing remote participation, design previews, and real-time feedback to improve esthetic predictability.
2. Occlusal design
  Bilateral balanced occlusion is recommended for complete dentures,^71^ including centric, protrusive, and lateral balanced occlusion. In centric occlusion design, occlusal contact analysis tools in the CAD software help visualize contact points for tooth positioning and optimal occlusion.
  For non-centric occlusion, a virtual articulator facilitates dynamic occlusal design.^66^ If personalized mandibular movement data is unavailable, an average-value articulator can simulate movements for virtual adjustments. When patient-specific parameters (e.g., condylar guidance, Bennett angle) are available, they can be imported to refine occlusal design. The software enables 3D adjustments of the entire arch, segments, or individual teeth, with real-time visualization of static and dynamic occlusal contacts.
  If diagnostic dentures function as occlusion rims for mandibular movement recording and jaw relation registration, and the trajectory is ideal, the patient’s actual mandibular movements can be imported to guide tooth arrangement and occlusal adjustments.

### Digital manufacture of complete dentures

#### Additive manufacturing

The primary technologies used in the additive manufacturing of digital complete dentures include stereolithography (SLA), digital light processing (DLP), material jetting, and powder bed fusion.^61^ In digital complete denture fabrication, 3D printing technology is applied in the following forms:^72–74^

1. Monolithic printing
  This method prints the denture base and artificial teeth as a single unit, eliminating the need for separate assembly and bonding. However, since both components are made from the same material, selecting a resin that balances strength, esthetics, and biocompatibility is essential. A single base shade is generally used, making it challenging to achieve natural pink and white esthetics. To enhance appearance, gingival staining is often applied after printing.
2. Split printing
  This method involves 3D printing of the denture base and artificial teeth separately. The printed teeth are bonded to the base during post-processing. This approach permits the use of different materials optimized for each component: denture base resin for the base and tooth-shade resin with superior color and translucency for the teeth. Such a strategy enhances the overall esthetics of the complete dentures. However, the additional bonding steps adversely affect the overall strength and accuracy of the definitive dentures.

#### Subtractive manufacturing

The technology used for subtractive manufacturing of complete dentures is computer numerical control (CNC) milling technology. This technology uses computer-controlled high-speed rotating tools to remove excess material from prefabricated material discs according to pre-programmed paths. Milling ultimately produces the desired shape of denture components.^75–78^ Based on the number of axes and functionality, milling machine may be classified as three-axis, four-axis, five-axis, and even higher-axis systems. High-axis milling machines enable more complex processing of curved surface.

The most commonly used CNC milling material in clinical practice is polymethyl methacrylate (PMMA) resin discs. The workflow involves fixing a pre-formed material disc onto the machine, after which the computer controls the trajectory and parameters of the tool according to the design data to achieve precise cutting. Milling be performed either as dry milling or wet milling (with a coolant). Wet milling is more commonly employed, as it enhances processing accuracy, improves surface quality, and extends the lifespan of the cutting tools.

Contemporary CNC milling for complete dentures is applied in the following forms:^75,79^

**Fig. 1:**
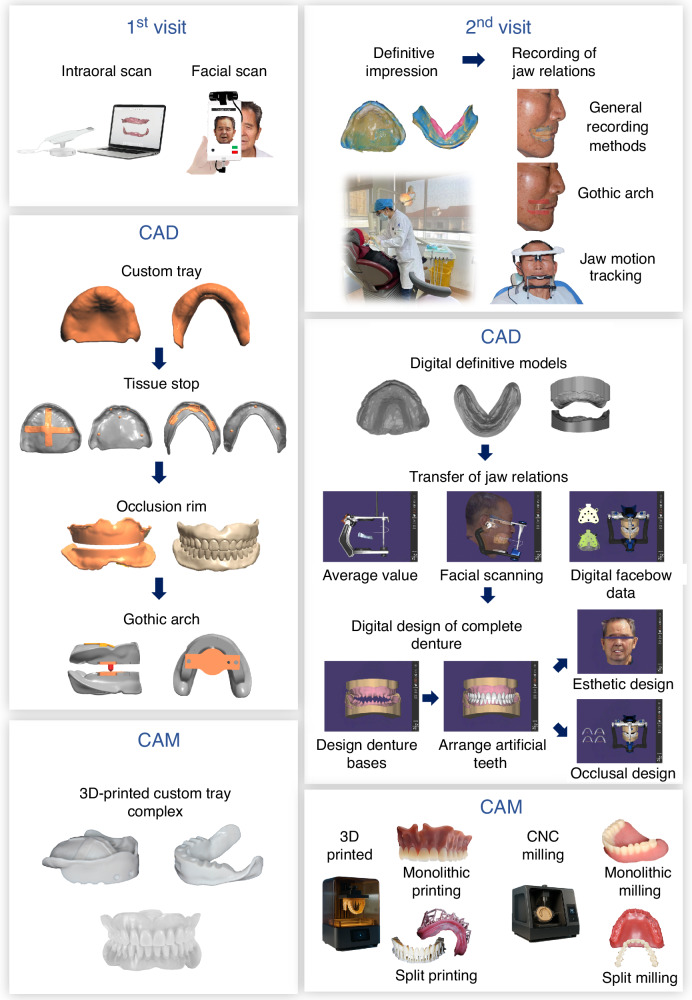
Schematic diagram of digital restoration of complete dentures

**Fig. 2:**
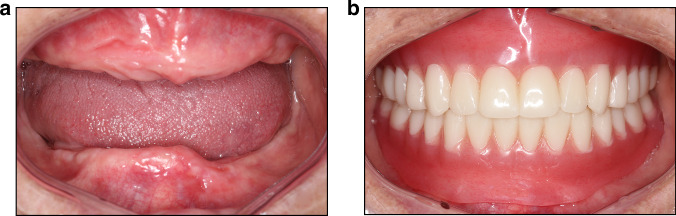
Digital restoration of complete dentures (**a**). Pre-treatment intraoral image (**b**). Post-treatment intraoral image

1. Monolithic milling
  This technique uses a single PMMA resin disc to mill both the denture base and artificial teeth. Based on the type of resin disc, monolithic milling may be classified into:
  *Milling with monochromatic resin discs*: Tooth-colored resin is generally used. During milling, the labial reduction amount is set, and gingival staining and modification are performed afterward using gingival-colored light-curing resin.
  *Milling with dichromatic resin discs (linear demarcation)*: These discs have a linear transition between pink and white resin, offering a basic simulation of the tooth-gingiva interface. Since the transition is fixed, the gingival contour may not fully align with the demarcation, and requires a reduction gap during milling. Post-processing includes shape correction and staining with gingival-colored light-curing resin.
  *Monolithic milling with dichromatic resin discs (curvilinear demarcation)*: Designed using extensive complete denture data, these discs feature a curved transition between teeth and gingiva, thereby eliminating the need for additional staining. The result is a natural, continuous gingival contour. However, design flexibility is limited, preventing modifications to gingival papilla shape or height.^79^
2. Split milling
  This method mills the denture base and artificial teeth separately. The two entities are bonded together during post-processing. High-strength PMMA is used for the base and resin composites are used for the teeth. The use of different materials optimized for each component enhances denture esthetics. However, bonding the teeth to the base requires a specialized denture adhesive, which may introduce errors during positioning. The choice of adhesive is crucial, as it directly affects the strength and durability of the final denture.^80–84^
  The general workflow of digital restoration of complete dentures is exhibited in Fig. 1, and the comparison of digital restoration of complete dentures, before and after treatment, is shown in Fig. 2.

### Follow-up and maintenance

Digital complete dentures are not a permanent restorative solution.^85^ Their longevity is affected by changes in the soft and hard tissues of a patient, as well as material wear via abrasion or fracture.

Short-term follow-ups after insertion are essential to check for pressure points, mucosal lesions, and evaluate of mastication, phonetics, and esthetics. Long-term maintenance requires regular recall visits every 6 to 12 months for comprehensive assessment of denture retention, stability, occlusion, base adaptation, tooth wear, and oral mucosal health. Based on these evaluations, relining or remaking may be necessary.^86^

### Other clinical scenarios

#### Transitioning from removable partial denture to complete denture

When transitioning a patient from a removable partial denture to a complete denture, digital technology can significantly streamline the restorative workflow, provided the existing removable partial denture is stable and can be reliably seated. The process begins with the use of an intraoral scanner to capture a digital edentulous model. Subsequently, digital models of the patient’s maxillary and mandibular arches with the existing denture in situ, along with the occlusal relationship, are obtained. Within the CAD software, the digital edentulous models are aligned with the digital models of the arches that include the existing denture. A diagnostic denture is then designed based on the existing denture and 3D-printed for evaluation. This diagnostic denture undergoes an intraoral try-in, after which definitive impression is taken, and adjustments are made to optimize esthetics and occlusal relationships. Finally, the definitive denture is fabricated using the digital data derived from the diagnostic denture. This approach leverages the existing denture’s design and fit to simplify traditional procedures, enhancing both efficiency and precision. However, the success of this method depends on the stability and accurate seating of the existing denture in the patient’s mouth. In cases where denture retention is insufficient, denture adhesive can be applied to improve stability during the workflow.

#### Complete denture re-fabrication

For patients requiring replacement of existing complete dentures, digital technology offers an efficient re-fabrication process. The existing denture serves as a custom tray to obtain a refined impression. Wax rims are added to the occlusal surfaces of the denture teeth, enabling intraoral adjustment to the occlusal plane and vertical dimension of occlusion. Subsequently, this refined impression is digital scanned. With the patient wearing the modified existing denture, the adjusted occlusal relationship is then captured through intraoral scanning. During the digital design phase, key elements of the previous denture, such as tooth morphology, arrangement, occlusal plane, and vertical dimension of occlusion, are thoroughly analyzed and referenced to guide the design and fabrication of the definitive prostheses. This workflow capitalizes on the valuable information embedded within the existing denture and integrates digital technologies to enhance traditional procedures. The result is a more accurate and patient-specific prosthetic solution that improves comfort, functionality, and overall treatment outcomes.

## Evaluation

### Accuracy evaluation of digital technology

#### Digital impression techniques

The accuracy of digital impressions is crucial for prosthesis quality and patient comfort. Research shows that indirect scanning produces models that are comparable to conventional gypsum models, offering high precision and reproducibility.^87,88^

Although direct intraoral scanning is used extensively in fixed prosthodontics, there are challenges to address in edentulous arch scanning.^39,89–91^ A systematic review of 39 studies confirmed that traditional border molding remains the predominant technique for digital denture production.^16^ Maxillary denture bases fabricated with border molding achieve better tissue adaptation and retention than those made via intraoral scanning.^91^ The major limitations of direct scanning are seen in areas with movable mucosa, such as the soft palate, sublingual area, and vestibular sulcus. These areas are critical for border sealing and retention.^40^ Despite these challenges, intraoral scanning deviations remain within 0.2 mm compared to conventional impressions, and under 0.9 mm relative to gypsum models. The miniscule deviations are indicative of clinically acceptable accuracy.^89–92^

#### Performance

Research highlights advantages of CAD/CAM technology in retention, stability, and precision. Retention force, measured using digital force gauges and universal testing machines, ranges from 13 to 20 N, with some studies showing better retention in digital dentures than conventional ones.^91,93–97^ However, a meta-analysis found that while conventional dentures had better retention than 3D-printed versions, milled dentures performed comparably.^17^ Impression techniques significantly impact retention; maxillary dentures made with border molding achieved forces up to 74 N, whereas those from intraoral scans had lower retention.^91^ Clinician surveys rate digital dentures higher than conventional ones,^98^ and subtractive manufacturing yields better retention than injection molding due to reduced polymerization shrinkage.^99^

Denture stability is crucial for resisting horizontal movement. However, it lacks standardized evaluation methods. While subjective assessments confirm digital dentures meet clinical stability requirements,^88,98^ further research is needed to develop objective evaluation criteria.

In vitro studies consistently show that subtractive manufacturing produces more precise denture bases than additive manufacturing.^15,100–103^ A study comparing seven fabrication systems found subtractive manufacturing had lower standard deviations, resulting in superior accuracy.^102,104,105^

Maxillomandibular relationship and occlusal accuracy significantly affect digital denture performance. Custom tray-occlusion rim complexes, existing dentures, or duplicate dentures are commonly used for recording jaw relations,^93–95,106^ enabling definitive impressions and jaw relation records to be completed in a single visit.^99,107,108^ Although gothic arch tracing is considered the gold standard for centric relation recording,^99,109^ no studies have validated the superiority of facebows, gothic arch tracers, or articulators in digital denture fabrication. Some research reports common occlusal errors, such as improper vertical dimension and anterior open bite. These errors are probably attributed to inaccuracies in jaw relation recording and the absence of clinical try-in steps.^110,111^

Masticatory function studies primarily assess occlusal force and efficiency. T-scan and occlusal force meters report maximum occlusal force for digital dentures ranging from 130 to 225 N.^98,112,113^ Findings on masticatory efficiency vary; some studies suggest improvements with digital dentures over existing conventional dentures, with no significant differences between fabrication methods.^98,114^ Others report no difference between digital and conventional dentures.^34^ Variability in study design, sample size, and evaluation methods may account for these discrepancies. This highlights the need for further high-quality research.

### Patient satisfaction

This is an important indicator of therapeutic outcomes. A meta-analysis found no significant difference in patient satisfaction between digital and conventional complete dentures,^17^ with similar findings for oral health-related quality of life (OHRQoL) across conventional, milled, and 3D-printed dentures.^93^ These results suggest that digital dentures offer comparable benefits in improving OHRQoL.

Digital fabrication reduces patient visits and chairside time, enhancing comfort and convenience.^99,115,116^ However, complications such as pain or tenderness are similar to those in conventional dentures.^106,109,111^ This finding indicates that digital workflow streamlines fabrication but does not eliminate discomfort.

Esthetics plays a major role in satisfaction. Some patients perceive 3D-printed dentures as less esthetically pleasing,^117^ though advancements in printing technology and materials are expected to improve this.^98^ Milled dentures may offer better esthetics due to material uniformity and predictability.

### Clinical benefits and cost

Apart from therapeutic outcomes, clinical benefits and costs are also critical aspects in evaluating digital complete dentures. Studies show that digital dentures require fewer clinical visits,^118,119^ improving efficiency and potentially reducing healthcare costs. Fabrication time is about one-third that of conventional methods, with significantly shorter laboratory processing.^120^ Digital workflow streamlines procedures, saving 58 to 233 min of clinical time.^115,117,121,122^ This results in better patient satisfaction and resource utilization. However, the high initial investment in digital equipment and staff training may impact cost-effectiveness. A thorough assessment of both short- and long-term benefits, along with associated costs, is essential for informed decision-making regarding digital denture integration into clinical practice.

## Conclusion and expectations

With the rapid advancement of digital technologies, complete denture restoration has entered a new era characterized by precision, efficiency, and enhanced patient-centered care. Digital protocols have reduced clinical chairside time and elevated the overall patient experience, marking clear progress compared to traditional methods. This expert consensus provided a structured framework and practical recommendations regarding indications, key procedural steps, quality control, and assessment methods to assist clinicians in optimizing the application of digital complete denture restoration techniques in clinical practice.

Looking forward, continuous advances in CAD/CAM software, equipment innovation, and novel materials will improve intraoral scanning accuracy for edentulous arches and lower barriers to adoption, expanding accessibility for both patients and clinicians. The integration of artificial intelligence (AI) and augmented reality (AR) is also anticipated to revolutionize CAD/CAM workflows and enable individualized esthetic customization, further enhancing clinical value of digital complete denture solutions.
